# Making eDNA more digestible: A pizza analogy for understanding false negatives and occupancy modeling

**DOI:** 10.1093/biosci/biag051

**Published:** 2026-05-30

**Authors:** Jonathan D Bolland, Nathan P Griffiths, Graham S Sellers, Bernd Hänfling, Lori Lawson Handley, Rosalind M Wright

**Affiliations:** Hull International Fisheries Institute, University of Hull, Hull HU6 7RX, UK; Hull International Fisheries Institute, University of Hull, Hull HU6 7RX, UK; EvoHull Evolutionary Biology and Environmental Genomics Group, University of Hull, Hull HU6 7RX, UK; Institute for Biodiversity and Freshwater Conservation, University of the Highlands and Islands, Inverness IV2 5NA, UK; EvoHull Evolutionary Biology and Environmental Genomics Group, University of Hull, Hull HU6 7RX, UK; EvoHull Evolutionary Biology and Environmental Genomics Group, University of Hull, Hull HU6 7RX, UK; Institute for Biodiversity and Freshwater Conservation, University of the Highlands and Islands, Inverness IV2 5NA, UK; EvoHull Evolutionary Biology and Environmental Genomics Group, University of Hull, Hull HU6 7RX, UK; Lake Ecosystems Group, UK Centre for Ecology and Hydrology, Lancaster Environment Centre, Bailrigg, Lancaster LA1 4AP, UK; Environment Agency, Rivers House, Threshelfords Business Park, Feering CO5 9SE, UK

**Keywords:** biodiversity, conservation, endangered species, molecular biology, policy

## Abstract

Environmental DNA (eDNA) sequencing from water samples has emerged as a promising and cost-effective approach to collect comprehensive freshwater biodiversity data. However, critics might highlight potential shortcomings, such as the possibility of false positives (detection of absent species) and false negatives (failing to detect a species that is present). Misconceptions and misunderstandings may also stem from the complexity of the scientific approach and technical language, with implications for decision-makers and potentially hindering conservation. In the present article, we propose an analogy of eating a pizza to simplify messaging and increase understanding of detection probability within typical eDNA metabarcoding workflows. The pizza represents a site, slices represent water samples, bites represent PCR replicates, toppings represent species and olives represent a low-abundance species. Overall, the pizza analogy provides a novel, lighthearted and memorable way to communicate complex eDNA workflows to a broad spectrum of biological scientists and practitioners.

Environmental DNA (eDNA) sequencing from water samples has emerged as a promising and cost-effective approach to collect comprehensive freshwater biodiversity data (Blancher et al. 2022). Several studies have shown increased sensitivity of eDNA-based monitoring for both entire fish communities and specific priority species when compared with traditional methods (Hänfling et al. 2016, Pont et al. 2018, Harper et al. 2019, Griffiths et al. 2020, Weldon et al. 2020). Substantial work has also been devoted to integrating eDNA-based monitoring into fish-based ecological status assessments in lakes (Willby et al. 2019) and rivers (Hering et al. 2018, Pont et al. 2018). Consequently, it is increasingly important that eDNA workflows and their outputs can be understood by non-molecular ecologists, including traditional fisheries scientists, managers and policy-makers.

In some instances, eDNA-based monitoring is lauded as a game changer (e.g., Carraro et al. 2020) and may lead to an overconfidence in its performance, whereas critics might highlight potential shortcomings, such as the possibility of false positives (detection of absent species; summarized by Cristescu and Hebert 2018), undermining its value and potential. In reality, neither perspective is likely to be completely true, and, like all sampling methods, eDNA has strengths and limitations that must be understood and handled accordingly (Darling 2020, Darling et al. 2020). For example, processes to monitor and mitigate false positives are well established, including the use of blanks or negative controls at each stage of the workflow (Ficetola et al. 2016), library preparation protocols aimed at minimizing cross-contamination (Bohmann et al. 2022), setting low-frequency reads thresholds (Hänfling et al. 2016), and adopting conservative occupancy modeling approaches (Stauffer et al. 2021). Notwithstanding, misconceptions often stem from the complexity of highly specialist scientific approaches, sometimes referred to as *black box science* (Morisette et al. 2021), and the use of technical language that can create a barrier to understanding (Stein et al. 2024). This is particularly problematic when environmental managers and decision-makers must interpret eDNA data without a clear framework for assessing its reliability and implications (e.g., Darling and Mahon 2011, Lodge 2024).

A key factor in assessing the presence or absence of a species is understanding the probability of false negatives (i.e., failing to detect a species that is actually present; Buxton et al. 2021). This is especially relevant for rare and elusive species that only occupy a subset of sites (i.e., low site occupancy) and may exist in low abundance when present (i.e., low detection probability). False negatives can arise from stochastic sampling processes at both the biological (water sampling) and the technical (PCR—polymerase chain reaction—amplification) stages of the workflow. In the biological stage, false negatives occur when no target species DNA is collected during water sampling, despite presence at the site. In the technical stage, false negatives occur when no target species DNA is present in the PCR replicate, despite being collected during water sampling. Increasing the number of biological samples and technical replicates can help mitigate the rate of false negatives (Ficetola et al. 2015), and multispecies site occupancy models attempt to address it (Doi et al. 2019, McClenaghan et al. 2020), with recent examples integrating read counts into such models (Fukaya et al. 2022; see table 1). However, without a thorough understanding of how false negatives can occur, non-molecular ecologists may erroneously conclude a species was absent from a site, potentially leading to flawed management decisions.

**Table 1.** A selected library of relevant multiscale occupancy modeling studies.

Study	Target taxa or context	Key findings
Nichols et al. (2008)	Vertebrates using multiple survey methods	Introduced multiscale occupancy modeling and method-specific detection probabilities
Schmidt et al. (2013)	Chytrid fungus	Demonstrated the need to separate water sampling and PCR detection in some eDNA occupancy models
Ficetola et al. (2015)	Soil earthworms and mammal sediment DNA	Applied occupancy models with replicated PCRs to estimate detection and false positives in eDNA and showed that high PCR replication is often needed for reliable detection
Fukaya et al. (2022)	Freshwater fish communities using eDNA metabarcoding	Developed a multispecies occupancy model that directly models sequence reads and informs survey design
Griffiths et al. (2025)	Freshwater fish communities using eDNA metabarcoding	Applied three-level occupancy modeling to quantify confidence in absence and proposed a framework for decision-making

In this article, we propose an analogy of eating a pizza to simplify messaging and increase understanding of detection probability within typical eDNA metabarcoding workflows. For simplicity, we focus on the two most common levels of replication, collecting multiple water samples from a site and carrying out multiple PCRs for each sample. The pizza represents a site, slices represent water samples, bites represent PCR replicates, toppings represent species and olives represent a low-abundance species (figure 1). We provide an example where three water samples were taken from a site, and each had three PCR replicates: Sample 1 is a positive result; the target species is present in two of the three PCR replicates. A single PCR replicate could have yielded a false negative (technical) despite presence in the sample. Sample 2 represents a biological false negative. The target species is absent in the sample despite presence at the site. Additional water samples may have yielded a positive. Sample 3 is a technical false negative. The target species is absent in all PCR replicates despite presence in the sample. Additional PCR replicates may have yielded a positive.

**Figure 1.**
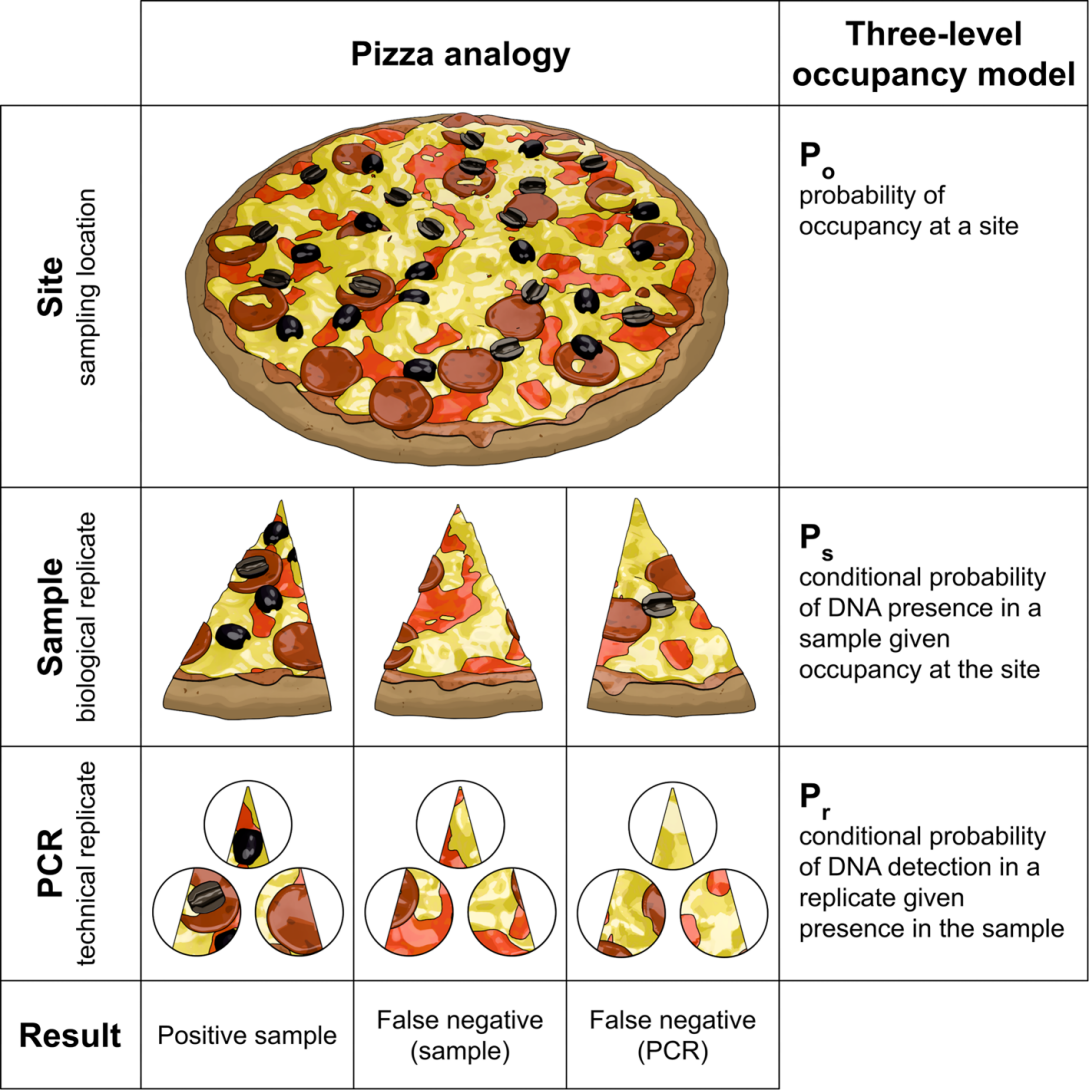
The pizza analogy. The pizza represents a site, slices represent water samples (biological replicate), bites represent PCR replicates (technical replicate) and olives represent a low-abundance species. Routes to olive detection (i.e., positive sample), and non-detection (i.e., false negatives during water sampling and PCR stages of the workflow) are presented, as is the terminology used during occupancy modeling.

In the example, the combination of three water samples (slices) and three PCR replicates (bites) could have accurately deduced the presence of the low-abundance species (olives) at the site (pizza); that is, the correct result was obtained. However, it also neatly demonstrates how a species can be missed at both water sampling and PCR stages of the workflow. This demonstration furthers the understanding of how the overall probability of occupancy at a site (*P_o_*), the conditional probability of DNA presence in a sample given occupancy at a site (*P_s_*), and the conditional probability of DNA detected in PCR replicates given presence in a sample (*P_r_*) are calculated (figure 1). In combination, these three probabilities allow the overall confidence in species absence at a site to be calculated according to the level of biological and technical replication (Griffiths et al. 2025). Indeed, following project specific applications and conference presentations, the authors have received strong positive feedback from both academic and non-academic stakeholders that the pizza analogy aids interpretation of eDNA workflows and assessments of species presence or absence. For example, it enables managers and funders to appreciate how a lack of detection may not mean a species is absent and why more sampling or lab work may be required.

The pizza analogy can also be extended, with collecting different volumes of water between samples represented by uneven size and shape of pizza slices, and the amount of DNA included in the PCR replicate would be represented in the present article as the size of each bite. It is also possible that low-abundance species are harder to detect with eDNA metabarcoding in complex communities (i.e., a false negative due to masking of target DNA via swamping from non-target DNA). For illustration, it may be harder to detect olives on a pizza with lots of toppings or flavors or where there is a dominant or overwhelming topping (e.g., chillies or pepperoni) than it would be on a simple pizza, such as a Margherita.

Overall, the pizza analogy provides a novel, lighthearted, and memorable way to communicate complex eDNA workflows. Specifically, the analogy simplifies different levels of occupancy modeling to enable a thorough understanding of how false negatives can occur and aid interpretation of how confident we can be in assessments of species absence. This has particular relevance to rare and conservation species and therefore enables non-specialist managers, regulators, and policy-makers to make more informed decisions. More generally, it is believed the pizza analogy will help make eDNA, which has emerged as a fundamental technology to quantify biodiversity, more digestible for a broad spectrum of biological scientists and practitioners.
